# Reproducibility of Digital Measurements on Soft and Hard Tissue

**DOI:** 10.3290/j.ohpd.c_1888

**Published:** 2025-03-20

**Authors:** Philipp Thißen, Mariam Mehr, Thomas Eger, James Deschner, Andreas Magnus Geyer

**Affiliations:** a Philipp Thißen Dentist, Bundeswehr Central Hospital Koblenz, Department of Dentistry-Periodontology, Koblenz, Germany; Department of Periodontology and Operative Dentistry, University Medical Center, Johannes Gutenberg-University Mainz, Mainz, Germany. Methodology and investigation, performed statistical evaluation, data curation, wrote original draft, revised draft.; b Mariam Mehr Dentist, Department of Periodontology and Operative Dentistry, University Medical Center, Johannes Gutenberg-University Mainz, Mainz, Germany. Methodology and investigation, performed statistical evaluation, data curation, wrote original draft, revised draft.; c Thomas Eger Periodontist, formerly Head of Department; Bundeswehr Central Hospital Koblenz, Department of Dentistry-Periodontology, Koblenz, Germany. Idea and hypothesis, methodology and investigation, wrote original draft, revised draft, project administration and funding acquisition.; d James Deschner Professor and Head of Department, Department of Periodontology and Operative Dentistry, University Medical Center, Johannes Gutenberg University Mainz, Mainz, Germany. Wrote original draft, revised draft, project administration.; e Andreas Magnus Geyer Dentist, Department of Periodontology and Operative Dentistry, University Medical Center, Johannes Gutenberg-University Mainz, Mainz, Germany. Wrote original draft, revised draft.

**Keywords:** digital dentistry, gingiva, intraoral scan, reproducibility

## Abstract

**Purpose:**

The aim of this proof-of-principle study was to investigate the reproducibility of digital hard- and soft-tissue measurements obtained using an intraoral scanner.

**Materials and Methods:**

Two consecutive digital scans of the maxilla and mandible of 20 subjects aged 18–58 years were captured with an intraoral scanner. Afterwards, the double scans of each subject were virtually matched by three different methods using a dental software program. Linear distances between defined hard- and soft-tissue points on the intraoral scans were measured for each individual. To assess the reproducibility of the measurements for each matching method, the corresponding linear distances of the first and second scans were compared using a paired t-test (p < 0.05). ANOVA (p < 0.05) was used for comparison of the three matching methods.

**Results:**

For both hard and soft tissue, the measured linear distances between the first and second scans did not differ statistically significantly. Furthermore, there were no statistically significant differences between the three matching methods for soft (p = 0.196) and hard (p = 0.963) tissue.

**Conclusion:**

Digital measurements of hard and soft tissue are reproducible using intraoral scans. Furthermore, all three matching methods are suitable for the superimposition of scans. However, possible inaccuracies may depend on the experience of the practitioner, the technical limitations of the systems used, and patient-related factors.

Intraoral scanners have become indispensable in dentistry. They can be used for various tasks and offer an alternative to analogue impression procedures related to precision and time savings.^6^ With the help of intraoral scanners, the gingiva can be captured without physical compression, which results in less inaccurate measurements compared to analog impressions.^5^ Determining the dimensions and colour of the gingiva can be an additional important diagnostic tool to identify possible pathologies, e.g., recessions, periodontitis, or peri-implantitis.^8,13^ Shrinkage and expansion of impression and plaster model materials can also be completely avoided. In addition, impression materials and costs can be saved when using an intraoral scanner. Taking digital impressions can be learned more easily and quickly and are less error-prone than conventional impressions, which requires extensive practice and experience.^3^ Although numerous studies have shown that digital impressions of hard tissues provide reliable data and are therefore reproducible, little is known about digital impressions of oral soft tissues.^2,9^ For periodontal, surgical and prosthetic treatments, soft tissue imaging can also play an important role in diagnosis and therapy. In addition, a meticulous data collection of 3-dimensional oral structures can provide an important basis for training neural networks.^14^ With increasing accuracy of modern scanners and continuous improvement of the software used for imaging and data processing, oral soft tissue should also be able to be imaged more precisely. The aim of this proof-of-principle study was to investigate the reproducibility of digital hard- and soft-tissue measurements obtained using an intraoral scanner.

## Material and Methods

### Intraoral Scanning

The Trios 3 intraoral scanner (3Shape; Copenhagen, Denmark) was used for the study. Before application, the scanner was calibrated according to the manufacturer’s instructions. A single dentist performed the intraoral scans at the Bundeswehr Central Hospital Koblenz, Germany. The scans were taken according to a scan path defined by the manufacturer and described in the instructions to ensure the quality and accuracy of the scans. The scan started in the mandible and proceeded continuously from the 3rd to the 4th quadrant, from the occlusal surfaces to the lingual and buccal/vestibular surfaces without interruption. In the maxilla, the path was continuous from the 2nd to the 1st quadrant, from the occlusal surfaces via the buccal/vestibular surfaces to the palatal surfaces, also without an intermediate stop. To map the occlusion of the jaws to each other, the maxilla and mandible were scanned in occlusion from the buccal side of the posterior teeth. The scans were done with as little overlap as possible to maintain accuracy and not overload the system with too much acquired data.

### Study Participants and Exclusion Criteria

Two consecutive digital scans of the maxilla and mandible of 20 subjects aged 18–58 years were captured using an intraoral scanner. Eleven of the subjects were female and nine were male, with ages ranging from 18 to 58 years.

Exclusion criteria for the subjects were:

severely altered soft tissueperiodontal attachment lossloss of Ramfjord teeth (16, 21, 24, 36, 41, 44)loss of central incisors in the maxilla and mandible.

### Digital Workflow

The double scans of each subject were virtually matched by three different methods using the “OrthoAnalyzer” evaluation program (3Shape; Copenhagen, Denmark). The entire digital workflow for evaluating the intraoral double scans was divided into three parts: a) segmentation of the individual teeth and construction of a virtual sagittal plane on each Ramfjord tooth; b) application of three different matching methods to compare the digital models of the double scans; and c) evaluation of the digital models and measurements.

First, teeth and gingiva were differentiated from each other in the software, and the center of rotation was determined for each tooth. The center of rotation was used to define a sagittal plane on which the local measurements could be performed.

Afterwards, the following three options provided by OrthoAnalyzer were used for the matching procedure: a) matching of the surface by 1 point between the two central incisors in the maxilla and mandible (matching 1); b) matching of the surface by 1 point on the respective Ramfjord tooth (matching 2); and c) matching of the surface by 3 points on the virtual jaw (matching 3).

**Fig 1 fig1:**
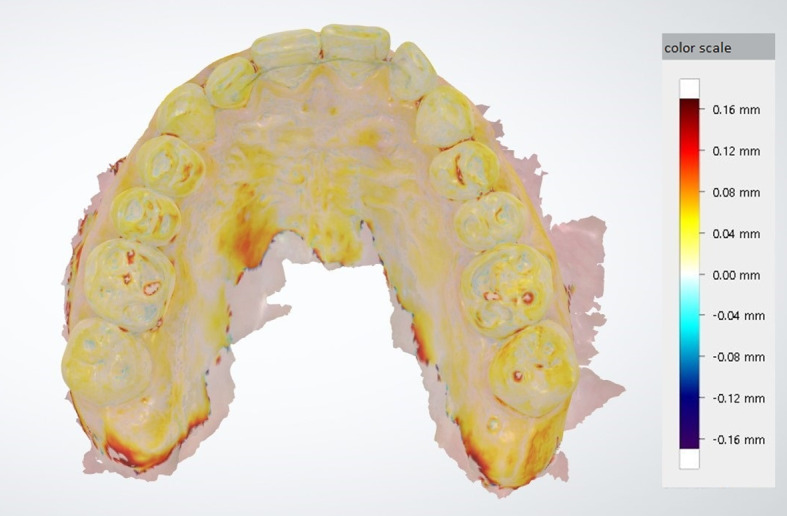
Superimposition of the initial and subsequent scan of a subject.

After applying one of the matching methods, the superimposition of the models was improved by an iterative closest-point algorithm (Fig 1). Variations of this algorithm are used to minimize distances between the point clouds of two 3D objects. This can further improve the initial manual superimposition. The three different matching methods are referred to as matching 1, matching 2 and matching 3 in the figures and rest of the manuscript for a better overview.

**Fig 2 fig2:**
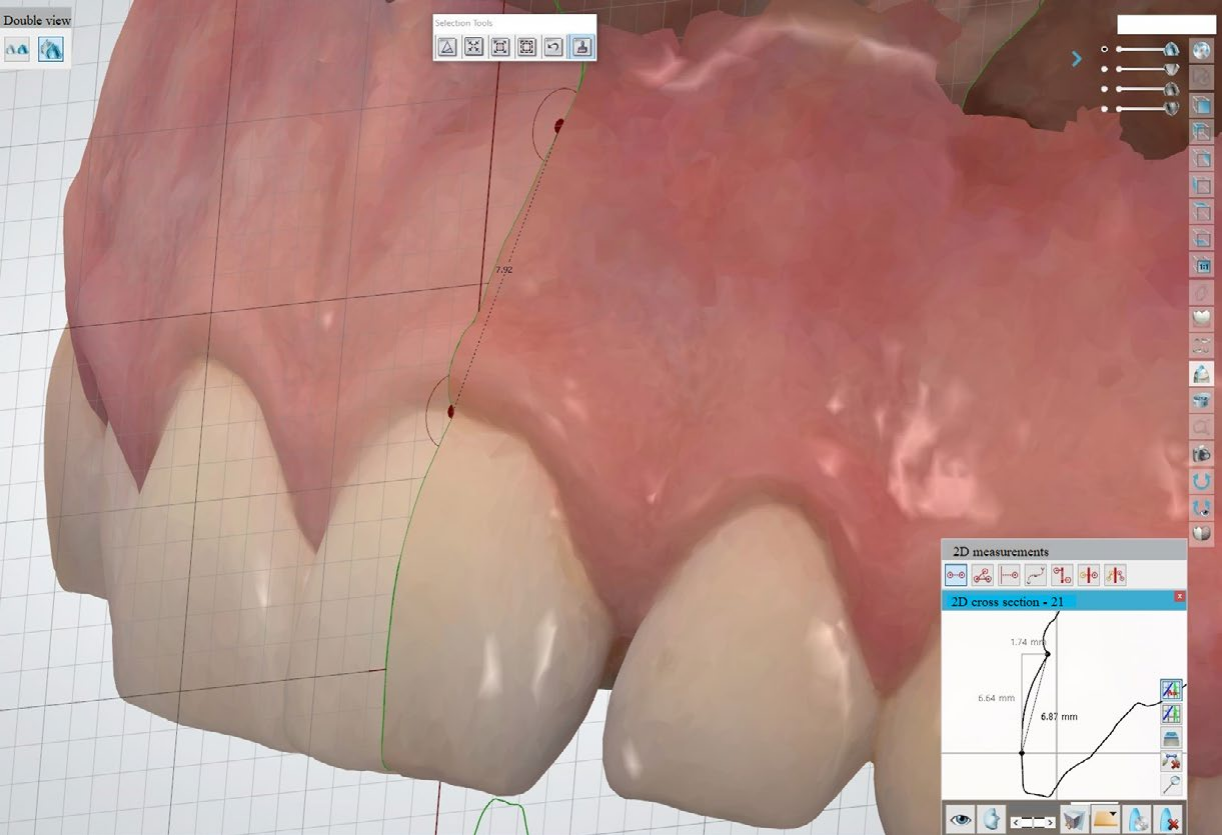
Measurement of the linear distances on hard and soft tissue in the maxilla.

**Fig 3 fig3:**
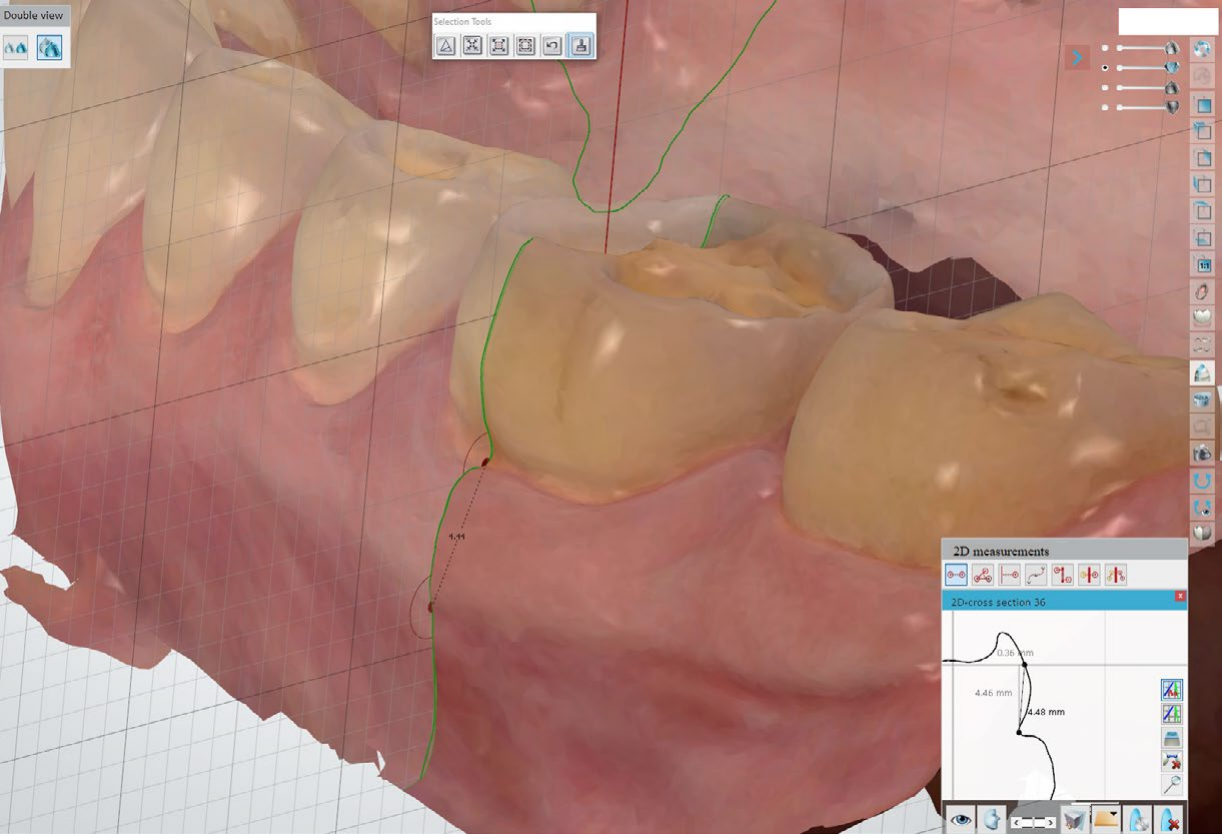
Measurement of the linear distances on hard and soft tissue in the mandible.

For the measurement of the linear distances on the hard tissue, two points were defined in the coordinate system of the generated sagittal plane (Figs 2 and 3). This plane is always automatically filled by the system with a geometric grid. The first point was defined at the intersection of the horizontal line of the geometric grid with the vestibular surface of the respective tooth (Figs 2 and 3). The second point was defined at the beginning of the sulcus margin on the sagittal plane (Figs 2 and 3). The distance from point 1 to point 2 was measured on both virtual models of each subject. The difference in the linear distances represents the discrepancies between the hard tissue measurements.

To assess the reproducibility of the soft tissue measurements, one point on the mucogingival junction was defined in the generated sagittal plane. The other point was located at the deepest position of the sulcus margin on the same plane (Figs 2 and 3). Staining the gingiva with Lugol’s iodine solution made the mucogingival junction more visible.

### Statistical Analysis

All tests were performed with the help of the SPSS software package (IBM SPSS Statistics 27.0.1; Armonk, NY, USA). Mean values and standard deviations were calculated. To assess the reproducibility of the hard and soft tissue measurements for each matching method, the corresponding linear distances of the first and second scans were compared with a paired t-test (p < 0.05). ANOVA (p < 0.05) was used for comparison of the three matching methods.

### Ethics Statement

The study was conducted on subjects of the Department of Dentistry-Periodontology at Bundeswehr Central Hospital Koblenz. All participants were military personnel. Written informed consent was obtained from all subjects involved in the study. The subjects were informed that participation in the study was voluntary, and that they could leave it at any time without consequences. In full compliance with ethical principles, the guidelines of the Declaration of Helsinki were followed, and the Regional Ethics Review of the Rhineland-Palatinate Medical Association in Germany (2019-14303) approved the study (16.05.2019). This study is registered in the German Register for Clinical Studies (DRKS00023185).

## Results

For matching 1, the differences between the two measurements for each subject were on average 0.06 mm ± 0.03 for the hard-tissue measurements and 0.11 mm ± 0.03 for the soft-tissue measurements. For matching 2, the differences between the two measurements were 0.07 mm ± 0.03 for hard tissue and 0.10 mm ± 0.03 for soft tissue. The differences between the two measurements for matching 3 were on average 0.07 mm ± 0.03 for hard tissue and 0.11 mm ± 0.02 for soft tissue.

**Fig 4 fig4:**
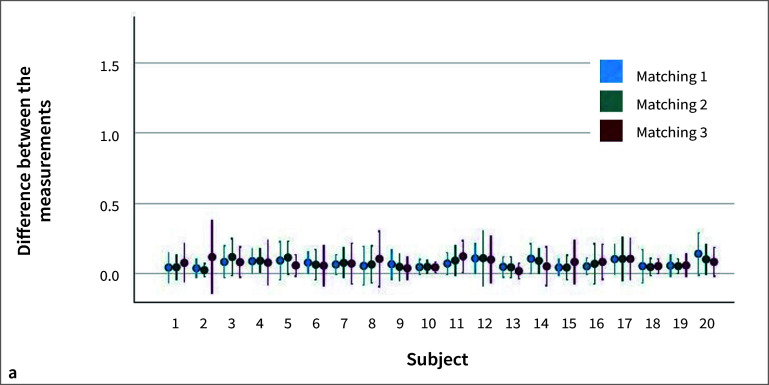

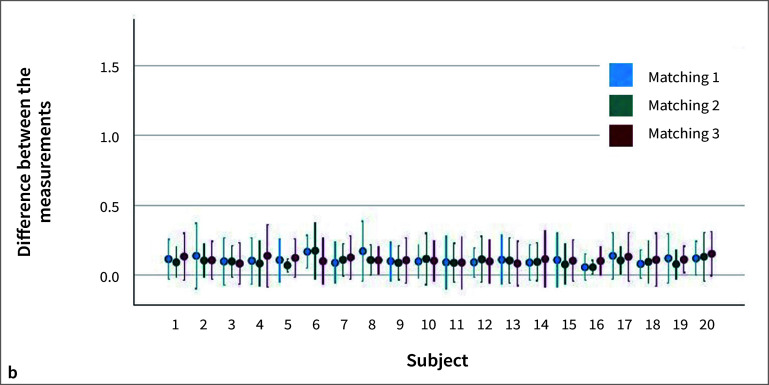
Differences between the measurements of the linear distances on hard (a) and soft (b) tissue (mm) per subject and matching method.

**Fig 5 fig5:**
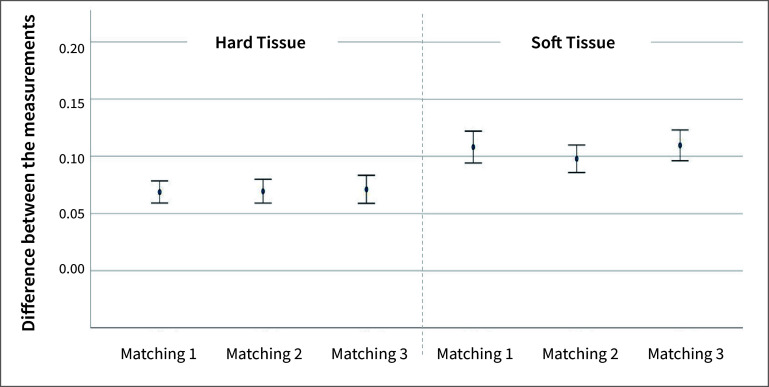
Differences between linear distance measurements on hard (left) and soft (right) tissue (mm) for all subjects combined per matching method.

The distances measured between the first and subsequent scan of the subjects after any of the three matchings did not differ statistically significantly for either hard or soft tissue (Figs 4a and 4b). Furthermore, no statistically significant differences were observed between the three matching methods for soft (p = 0.196) and hard (p = 0.963) tissue (Fig 5).

## Discussion

With all three matching methods provided by the software, the hard and soft tissue could be reproducibly measured without statistically significant differences in the measured distances. The deviations in precision were within a clinically acceptable range for both tissue types.^¹⁵^ Since this was a feasibility study, 20 subjects were scanned initially. For follow-up studies, it would be useful to increase the number of subjects to compensate for the variability of the study participants.

The precision of the measured distances after matching the 3D models was critical in assessing the reproducibility of digital measurements on intraoral hard and soft tissue. The precision and trueness of the scanner for the full arch has been investigated previously, with values of approximately 50 μm for trueness and 100 μm for precision.^7,12,15^ These precision values are reflected in the local distances we measured for hard and soft tissue in the present study.

Since digital images captured by the scanner are combined into a 3D model by the scanner software, inaccuracies also occur during this process. Improvements in precision on the hardware side can be achieved by increasing the number of images per second, increasing the resolution of the images, and reducing the size of the scan head to facilitate data acquisition. The software also has a major influence on the quality of the calculated 3D models, both for creating the models and for matching.^17,19^


Since the software provides an algorithm for matching, all scans could be matched without much effort. At the same time, however, the software does not allow any adjustments to the provided algorithms. Depending on the data, the superimposition of the models can be improved, for example, by defining relevant areas, combining point-to-point and point-to-plane matching algorithms, or by adjusting the iterations of the algorithm.

A critical factor in image quality is the absence of saliva on the surface of hard and soft tissue. Although intraoral scanning allows suction and drying of the tissue surface parallel to the scan, a completely dry oral situation cannot be achieved in vivo. The influence of saliva alone has been measured to cause a deviation of up to 13% of the original surface area on dental models.^4^


The characteristics of the soft tissue can also affect the accuracy of the images. Deeply inserted buccal or labial frenula with unattached alveolar mucosa generally do not allow clinically satisfactory images. However, in the area of the gingiva, satisfactory image quality can usually be achieved with an intraoral scanner due to the dimensional stability provided by the adhesion of the tissue to the periosteum and the high proportion of collagen fibres.^10^


Staining the mucosa with Lugol’s iodine solution could make it easier to distinguish between the alveolar mucosa and the gingiva. Automated segmentation based on the staining could be useful for future studies, but to the best of our knowledge, it is not an available function of the software we used or of comparable programs.

Practiced handling of the hardware used can also improve the accuracy of the scans.^1,11,18^ Depending on the acquisition time of a full scan, the software will produce better results. While too short an acquisition time may provide too little data to successfully capture the tissue, too many images can reduce the accuracy of the scans.^9^


## Conclusion

The results of the present study show that an intraoral scanner in combination with the appropriate software can measure hard- and soft-tissue structures with good reproducibility. As the comparison of the different matching methods showed no statistically significant differences, all three procedures can be recommended. In addition to the technical limitations, the experience of the practitioner, the scanning strategy used, and patient-related factors such as saliva flow and mucosa type can influence the accuracy of the intraoral scan.

## Acknowledgments

Special thanks for the repeated German–English translations and formal help go to Anika Noto Eger, Toronto, Canada. The study was funded by the Bundeswehr Medical Service Academy, Munich, Germany. The funders had no role in the design of the study, in the collection, analysis, or interpretation of data, in the writing of the manuscript, or in the decision to publish the results. The opinions expressed in this article are those of the authors. They cannot be construed as reflecting the views of the Bundeswehr Medical Service, the Bundeswehr at large, or the German Ministry of Defence.

## References
